# Supplementary material for: Pediatric Malaria with Respiratory Distress: Prognostic Significance of Point-of-Care Lactate
Source: Microorganisms. 2023 Apr 2;11(4):923. doi: 10.3390/microorganisms11040923 (PMC10145304; doi:10.3390/microorganisms11040923)
Supplement: Supplementary file 1 [file microorganisms-11-00923-s001.zip › microorganisms-2304191-supplementary.pdf]

**Table S1.** Number of patients from three primary studies contributing to the pooled secondary analysis, with assessment of completeness of data from each study

| | Study 1 (N=43)[1] | Study 2 (N=601)[2] | Study 3 (N=680)[3] | Pooled total (N=1324) |
|---|---|---|---|---|
| ***Demographics*** | | | | |
| Female sex | 43 (100) | 599 (100) | 680 (100) | 1322 (100) |
| Age [years], median (IQR) | 43 (100) | 601 (100) | 680 (100) | 1324 (100) |
| ***History*** | | | | |
| Cough | 43 (100) | 601 (100) | 680 (100) | 1324 (100) |
| Difficulty breathing | 43 (100) | 600 (100) | 680 (100) | 1323 (100) |
| Lethargy | 43 (100) | 600 (100) | 680 (100) | 1323 (100) |
| Convulsions | 43 (100) | 601 (100) | 680 (100) | 1324 (100) |
| Unable to feed/drink | 43 (100) | 599 (100) | 680 (100) | 1322 (100) |
| Vomiting | 43 (100) | 598 (100) | 680 (100) | 1321 (100) |
| Diarrhea | 43 (100) | 599 (100) | 676 (99) | 1318 (100) |
| ***Physical examination findings*** | | | | |
| Weight [kg], median (IQR) | 43 (100) | 593 (99) | 679 (100) | 1315 (99) |
| Length/height [cm], median (IQR) | 0 | 595 (99) | 674 (99) | 1269 (96) |
| Heart Rate (bpm), median (IQR) | 43 (100) | 589 (98) | 680 (100) | 1312 (99) |
| Respiratory rate (bpm), median (IQR) | 43 (100) | 584 (97) | 680 (100) | 1307 (99) |
| Oxygen saturation (%), median (IQR) | 43 (100) | 592 (99) | 680 (100) | 1315 (99) |
| Temperature | 43 (100) | 591 (98) | 680 (100) | 1314 (99) |
| Altered level of consciousness | 43 (100) | 601 (100) | 680 (100) | 1324 (100) |
| Capillary refill time | 43 (100) | 592 (99) | 680 (100) | 1315 (99) |
| Chest indrawing | 43 (100) | 601 (100) | 680 (100) | 1324 (100) |
| Wheeze | 0 | 0 | 680 (100) | 680 (51) |
| Stridor | 0 | 0 | 680 (100) | 680 (51) |
| Danger signs | 43 (100) | 599 (100) | 679 (100) | 1321 (100) |
| ***Investigations*** | | | | |
| Hemoglobin | 43 (100) | 359 (60) | 408 (60) | 810 (61) |
| Creatinine | 42 (98) | 224 (37) | 0 | 266 (20) |
| Parasite density | 43 (100) | 479 (80) | 333 (49) | 855 (65) |
| ***Treatment*** | | | | |
| Artesunate | 43 (100) | 601 (100) | 680 (100) | 1324 (100) |
| Quinine | 43 (100) | 601 (100) | 680 (100) | 1324 (100) |
| Artemether injection | 43 (100) | 601 (100) | 680 (100) | 1324 (100) |
| Artemether-lumefantrine (oral) | 43 (100) | 601 (100) | 680 (100) | 1324 (100) |
| Supplemental oxygen | 0 | 601 (100) | 680 (100) | 1281 (97) |
| ***Outcome*** | 43 (100) | 594 (99) | 680 (100) | 1317 (99) |

[1]Study 1 was a randomized controlled trial of inhaled nitric oxide as adjunctive treatment for severe malaria (n=180). In this secondary analysis, we included patients from the placebo group, with respiratory distress, with a documented lactate measurement (n=49).
[2]Study 2 was a prospective observational study of children with febrile illness presenting to a single regional referral hospital. In this secondary analysis, we included patients who tested positive for malaria, with respiratory distress, with a documented lactate measurement (n=603).
[3]Study 3 was a prospective study of children with hypoxemia presenting to 20 rural hospitals or health centres. In this secondary analysis, we included patients with fever who tested positive for malaria, with respiratory distress, with a documented lactate measurement (n=680 from 20 sites).

**Supplemental Text.** Estimation of proportion of lactate production by parasites

To estimate the relative production of lactate from host tissues and from *Plasmodium* parasites, we followed the method of Possemiers *et al.* [28]. We used estimates from the published literature for total lactate production in children with severe malaria [71], lactate production from parasites, based on *in vitro* data [27], blood volume as a function of body weight for children [72], and ratio of sequestered to circulating parasites [65]. Individual patient data from the present study was used for the body weight and parasite density. We used the semi-quantitative "plus" system to estimate the circulating parasite density, following the method of Kosack *et al.* [43].

***Lactate from host tissues:***

Total lactate production in children with severe malaria: 94 μmol/kg/min [71]
Weight (kg): median 9.0 kg (IQR 7.7 to 12)
Total lactate production rate:

$$= 94\ \mu\text{mol/kg/min} \times \frac{60\ \text{min}}{\text{hour}} \times \frac{24\ \text{hour}}{\text{day}} \times \frac{1\ \text{mmol}}{1000\ \mu\text{mol}} \times wt[\text{kg}]$$

**median 1200 mmol/day (IQR 1000-1600)**

***Lactate from parasite source:***

Lactate production by iRBCs: $0.524\times10^{-12}$ mol/iRBC/day [27]
Blood volume: estimated 80mL/kg body weight [72]
median 720mL (IQR 620-960)
Circulating parasite density: median 500 parasites/uL (IQR 50 to 5000)

Total circulating parasites:

$$= (circulating\ parasite\ density\ [\text{iRBC}/\mu\text{L}]) \times (blood\ volume)$$

$$= (circulating\ parasite\ density\ [\text{iRBC}/\mu\text{L}]) \times \left(\frac{80\ \text{ml}}{\text{kg}} \times wt[\text{kg}]\right) \times \frac{1000\ \mu\text{L}}{\text{mL}}$$

median $0.44\times10^{9}$ iRBC (IQR 0.036 to 3.6)

Ratio of sequestered to circulating parasites:
Non-fatal cases: 17 [65]
Fatal cases: 30 [65]
Total body parasites:

$$= (total\ circulating\ parasites) + (sequestered\ parasites)$$

$$= (total\ circulating\ parasites) + \left(circulating\ parasites \times \frac{sequestered\ parasites}{circulating\ parasites}\right)$$

median $8.6\times10^{9}$ iRBC (IQR 0.65 to 67)

Parasite lactate production rate:

$$= 0.524 \times 10^{-12}\ \text{mol/iRBC/day} \times (total\ body\ parasites\ [\text{iRBC}]) \times \frac{1000\ \text{mmol}}{\text{mol}}$$

**median 4.5 mmol/day (IQR 0.34-35)**

***Proportion of lactate from parasite:***

$$= \frac{parasite\ lactate\ production\ rate}{total\ lactate\ production\ rate}$$

**median 0.28% (IQR 0.028-2.8)**
